# Comprehensive analysis of differentially expressed profiles of lncRNAs and circRNAs with associated co-expression and ceRNA networks in bladder carcinoma

**DOI:** 10.18632/oncotarget.9706

**Published:** 2016-05-30

**Authors:** Mengge Huang, Zhenyu Zhong, Mengxin Lv, Jing Shu, Qiang Tian, Junxia Chen

**Affiliations:** ^1^ College of Clinical Medicine, Southwest Medical University, Luzhou 646000, China; ^2^ The First Clinical College, Chongqing Medical University, Chongqing 400016, China; ^3^ Department of Cell Biology and Genetics, Chongqing Medical University, Chongqing 400016, China; ^4^ Department of Clinical Laboratory, The Affiliated Hospital of Southwest Medical University, Luzhou 646000, China; ^5^ Department of Cell Biology and Genetics, Southwest Medical University, Luzhou 646000, China

**Keywords:** lncRNA, circRNA, ceRNA, microarray, bladder carcinoma

## Abstract

Accumulating evidences indicate that long noncoding RNAs (lncRNAs) and circular RNAs (circRNAs) play important roles in tumorigenesis. However, the mechanisms remain largely unknown. To explore lncRNAs and circRNAs expression profiling and their biological functions in bladder cancer, we surveyed the lncRNA/circRNA and mRNA expression profiles of bladder cancer and para-cancer tissues using microarray for four patients. Thousands of significantly changed lncRNAs and mRNAs as well as hundreds of circRNAs were identified. Five dysregulated lncRNAs and four mRNAs were confirmed by quantitative real-time PCR in 30 pairs of samples. GO and KEGG pathway enrichment analyses were executed to determine the principal functions of the significantly deregulated genes. Further more, we constructed correlated expression networks including coding-noncoding co-expression (CNC), competing endogenous RNAs (ceRNA), cis regulation, lncRNAs-transcription factor (TF)-mRNA with bioinformatics methods. Co-expression analysis showed lncRNA APLP2 expression is correlated with apoptosis-related genes, including PTEN and TP53INP1. CeRNA network inferred that lncRNA H19 and circRNA MYLK could bind competitively with miRNA-29a-3p increasing target gene DNMT3B, VEGFA and ITGB1 expressions. Moreover, the nearby genes pattern displayed that overexpressing ADAM2 and C8orf4 are cis-regulated by lncRNA RP11-359E19.2, involving in progression of bladder cancer. In addition, lncRNAs-TF-mRNA diagram indicated that lncRNA BC041488 could trans-regulate CDK1 mRNA expression through SRF transcription factor. Taken together, these results suggested lncRNAs and circRNAs could implicate in the pathogenesis and development of bladder cancer. Our findings provide a novel perspective on lncRNAs and circRNAs and lay the foundation for future research of potential roles of lncRNAs and circRNAs in bladder carcinoma.

## INTRODUCTION

Bladder cancer (BC) is the most popular urologic cancer with distinctive morbidity and mortality throughout the world, and is the seventh most prevalent cancer in males. According to data of Global Cancer Statistics, about 440,000 new cases of bladder cancer are diagnosed while 130,000 patients died from it around the world every year. In China, bladder cancer has the highest incidence in all urinary system tumors and the mortality increased significantly during the past decades. High recurrence rate is one representative characteristic of bladder cancer [1, 2]. This highlights the pressing need for new biomarkers for a more precise prediction of bladder cancer recurrence and cancer therapy.

Approximately 5%-10% of the sequence is transcribed in human genome. Among transcripts, about 10-20% are the protein-coding RNAs, and the rest 80%-90% are non-protein-coding RNAs. Long non-coding RNA (lncRNA) is a kind of non encoding RNA longer than 200nt [3]. Recently, more and more evidences have showed that lncRNAs play a crucial part in tumorigenesis and progression of tumor. They could regulate gene expression by cis and trans means [4, 5]. Recently, researches showed that dyregulated lncRNAs were implicated in the occurrence and development of bladder cancer. Searching a lncRNA signature might be of clinical value for diagnosis and prognosis of bladder cancer [6]. However, to date, little is known about a genome-wide expression and function analysis of lncRNAs in BC.

Recently, researchers paid attention to a new class of RNA that is single-stranded, covalently closed circular molecules in the cells. Although the functions of circRNAs remain largely unknown, it has been demonstrated that the circRNA CDR1as (ciRS-7) and SRY could harbor specific miRNAs as miRNA sponges. Resistant to exonuclease, circRNAs are stable molecules in cells. This characteristic makes the possibility of circRNAs as biological markers in clinical samples. It is surmised that circRNAs could regulate gene expression at epigenetic level by microRNA (miRNA) sponges. However, the number of circRNAs as miRNA sponges is unclear so far. Whether these circular isoforms implement any other biological function is still poorly understood [7–9]. As far as we know, there is no research about the functional roles of circular RNA to be reported in bladder cancer.

CircRNAs could negatively regulate the activity of miRNAs as miRNA sponges by competing endogenous RNA (ceRNA) network. ceRNAs include pseudogene transcripts, lncRNAs, circRNAs and mRNAs, these transcripts can compete for the same microRNA response elements (MREs) to regulate mutually. ceRNAs implicate in the tumorigenesis of some kinds of cancers, for example, liver cancer, lung cancer, gastric cancer, breast cancer, prostate cancer, etc. Theoretically, any RNA transcript with MREs might act as ceRNA. If the balance of ceRNA intricate network is disturbed, it could lead to cancer. Exploring for this RNA interaction would offer novel perspective for the molecular mechanisms of carcinogenesis and furnish a new clue for cancer therapy [10, 11]. However, no information on bladder cancer ceRNA has been reported to date.

The molecular mechanisms underlying the non-coding RNAs role in carcinogenesis and progression of urinary bladder cancer remain largely unclear. Accordingly, in order to understand the molecular profiles of non-coding RNAs for bladder cancer, in the present study, we investigate the differentially expressed patterns of lncRNA, mRNA and circRNA in bladder cancer using microarray. The significant differential expressions of representative mRNAs and lncRNAs were further confirmed using qRT-RCR. Subsequently, we not only acquired associated pathways and gene ontology items, but also delineated comprehensive functional landscapes of the coding-noncoding co-expression (CNC), ceRNAs, lncRNAs' nearby coding and TF-lncRNAs enriched with mRNA network for the first time with specific bioinformatics approaches in bladder cancer. Furthermore, our data showed that several lncRNAs and circRNAs could serve as diagnostic and therapeutic markers for BC. Finally, we predicted the functions of the lncRNAs and circRNAs by investigating these differentially expressed lncRNAs, circRNAs and mRNAs' co-expression networks. Our findings might illuminate the novel mechanism of bladder carcinoma pathogenesis and provide new targets for BC.

## RESULTS

### Differentially expressed lncRNA, mRNA and circRNA profiles by microarray

**Figure 1:**
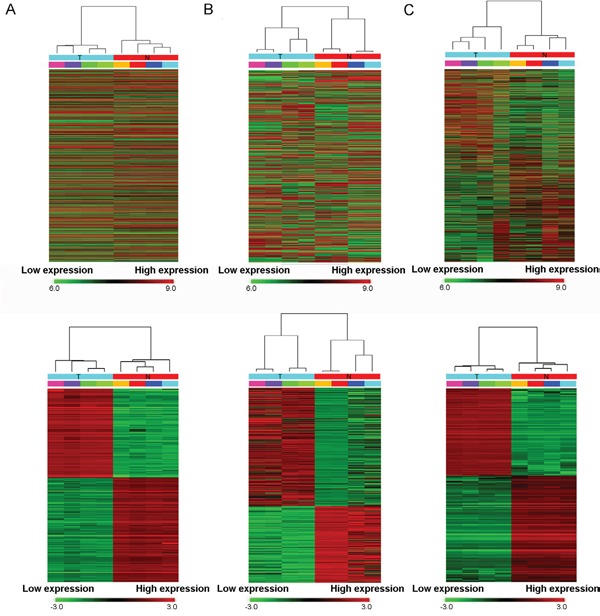
Heat map showing expression profiles of lncRNAs (A), circRNAs (B) and mRNAs (C) The upper maps are based on expression values of all expressed lncRNAs, circRNAs, and mRNAs detected by microarray probes respectively. While, the bottom maps correspond to normalized expression values of significantly changed lncRNAs, circRNAs, and mRNAs with fold change ≥2.0, P < 0.05 and FDR < 0.05. The expression values are depicted in line with the color scale. The intensity increases from green to red. Each column represents one sample, and each row indicates a transcript. T: tumor tissues. N: normal tissues.

High throughput microarray is an efficient approach for investigating the biological function of RNAs. As shown in the top row of Figure 1, the microarray probes have detected thousands of transcripts in bladder cancer and normal tissues. And as shown in the bottom row, a total of 4155 lncRNAs and 4416 mRNAs were detected to be differentially expressed with fold change ≥2.0, P < 0.05 and FDR < 0.05. Among them, 2045 and 2110 lncRNAs were upregulated and downregulated, meanwhile, 2472 and 1944 mRNAs were upregulated and downregulated (fold change ≥2.0, P < 0.05 and FDR < 0.05) in four BC tissues compared with controls, respectively. And that, the microarray expression profiles contained a total of 3243 circRNAs were dysregulated in BC. Filtering analysis of fold change ≥2.0, P < 0.05 and FDR < 0.05 identified remarkably differentially expressed 469 circRNAs between carcinoma and normal tissues, among them 285 up-regulated and 184 down-regulated. 345 lncRNAs displayed fold change ≥10, including 127 up-regulated lncRNA and 218 down-regulated lncRNAs. RP11-436F21.1 (fold change: ~236) was the most upregulated lncRNA. H19 is one of the most up-regulated lncRNAs in BC compared with the adjacent noncancerous tissue (fold change: ~30). Overview of coding gene profile showed that 75 mRNAs had fold change ≥10 (up: 57; down: 18). Hierarchical clustering showed that lncRNA, circRNA and mRNA expression patterns among samples were distinguishable. The data suggested that the expressions of lncRNAs, circRNAs and mRNAs in BC tissues are different from those in matched non-tumor tissues (Figure 1).

**Figure 2:**
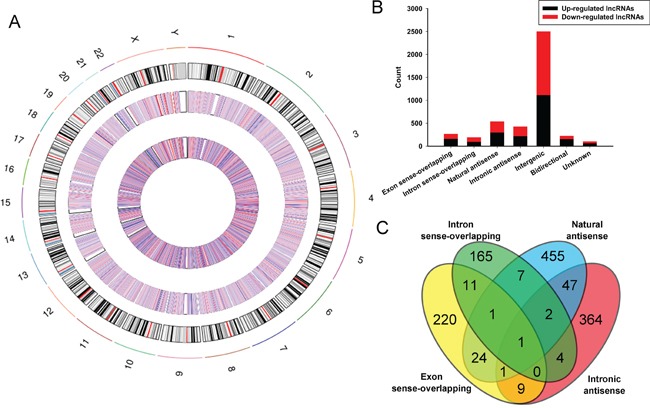
Identification of differentially expressed lncRNAs in bladder carcinoma tissues **A.** Circos plot showing lncRNAs on human chromosomes. The outermost layer of circos plot is chromosome map of the human genome, black and white bars are chromosome cytobands, red bars represent centromeres. The increased or decreased lncRNAs have been marked in red or blue bars, respectively. The larger inner circle represents all target lncRNAs dectected by microarray probes, and the smaller inner circle indicates the significantly differentially changed lncRNAs with fold change≥2.0, P < 0.05 and FDR < 0.05. **B.** Types and counts of differentially regulated lncRNAs detected by microarray (fold change ≥2.0, P < 0.05 and FDR < 0.05). The lncRNAs are classified into 6 types according to the relationship and genomic loci with their associated coding genes. One lncRNA may be associated with several mRNAs for disparate relationships. **C.** The Venn diagram presents the overlapping of relationships and the numbers indicate the lncRNA counts. While, the amounts of each lncRNA types are totalized in the bar chart showing the up and down regulated lncRNAs respectively.

These lncRNAs are widely distributed in all chromosomes covering sex chromosomes X and Y (Figure 2A). Among the dysregulated lncRNAs, in the light of their relation with protein-coding genes, the lncRNAs were classified into six categories: 6.3% were Exon sense-overlapping, 4.5% were intron sense-overlapping, 12.6% were natural antisense, 10.1% were intronic antisense (Figure 2B). These four categories have a certain number of cross (Figure 2C). 58.9% were intergenic, 5.3% were bidirectional and 2.4% were unknown. While, intergenic lncRNAs stand for the largest category (58.9%) in all differentially expressed lncRNAs, and we detected greater percentages of intergenic lncRNAs in both upregulated (25.1%) and downregulated lncRNAs (33.8%) in BC compared with control.

### Validation of deregulated lncRNAs and mRNAs

**Figure 3:**
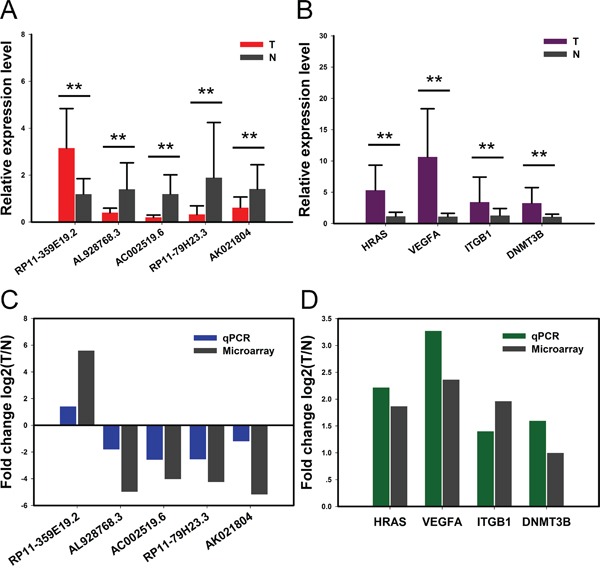
Validation for the expression of significant transcripts by quantitative RT-PCR The relative expression levels of five lncRNAs **A.** and four mRNAs **B.** are shown comparing tumor tissues (T) and normal tissues (N). Data are presented as mean ±SD, n=30. **P<0.01. **C-D.** Comparison between qPCR results and microarray data revealing a good correlation of such two methods. The heights of the columns represent the fold changes (log2 transformed) computed from the qPCR and microarray data.

Five lncRNAs and four mRNAs were chose for verification of the microarray results in 30 pairs of samples by quantitative real-time PCR. qRT-PCR assay showed that the expression of lncRNA RP11-359E19.2 was upregulated, whereas AL928768.3 and AC002519.6 as well as RP11-79H23.3 and AK021804 were downregulated (Figure 3A). Meanwhile, 4 target mRNAs HRAS, VEGFA, ITGB1 and DNMT3B were all upregulated in BC compared with control, respectively (Figure 3B). The result is consistent with the microarray assay (Figure 3C and Figure 3D). Hence, the qRT-PCR data verified the veracity of microarray results. The finding provides valid evidence that these lncRNAs and mRNAs could be implicated in pathogenesis of BC.

### Delineation of gene ontology (GO) and KEGG pathway analysis

**Figure 4:**
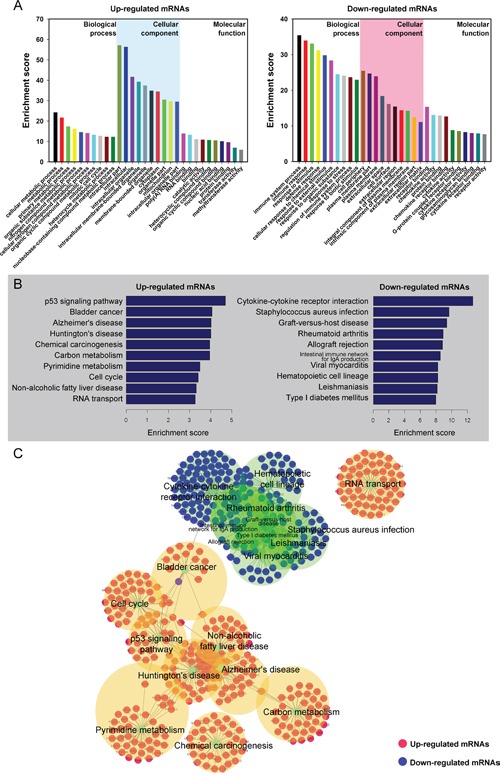
Gene Ontology (GO) and pathway analysis **A.** Go annotation of up and down regulated mRNAs with top ten Enrichment score covering domains of biological processes, cellular components and molecular functions. **B.** KEGG pathway enrichment analysis of up and down regulated mRNAs with top ten Enrichment score. **C.** The link and overlapping of associated molecules among significant pathways.

It was well known that lncRNAs could regulate the neighboring and overlapping coding gene expressions. Therefore, lncRNAs execute functions that might be embodied in related mRNA genes. Gene ontology (GO) enrichment analysis of significantly differentially expressed mRNAs can reveal the role of obviously differentially regulated lncRNAs. Our data showed that the upregulated mRNA, associated to biological processes, were cellular metabolic and mitotic cell cycle. Meanwhile, the downregulated transcripts were most relevant to immune system process, response to stimulus and immune response. Crucially, the mitotic cell cycleand methyltransferase activity GO terms correlate with cancer, which is important in controlling cell proliferation and gene expression (Figure 4A).

KEGG pathway enrichment analysis for significantly differentially expressed mRNAs is devised to comprehend pathways and molecular interaction related to genes. Our data showed that 10 pathways associated to upregulated mRNAs and 10 related to downregulated mRNAs. The p53 signaling pathway and Bladder cancer were the top pathways in upregulated protein-coding genes, whereas, the top enriched KEGG pathway was Cytokine-cytokine receptor interaction for downregulated transcripts (Figure 4B and Figure 4C). The result suggests that these pathways might contribute significantly to the pathogenesis and development of BC.

**Figure 5:**
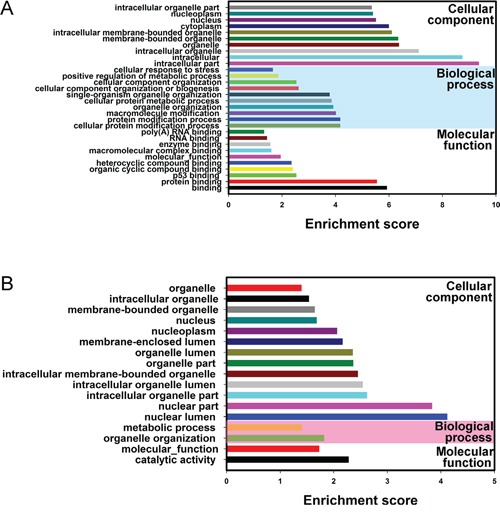
Gene Ontology (GO) analysis of the genes producing differentially expressed circRNAs Go annotation of the linear counterparts of up-regulated circRNAs **A.** and down-regulated circRNAs **B.** with top ten Enrichment score covering domains of biological processes, cellular components and molecular functions. Both up and down regulated circRNAs are significantly changed with fold change ≥2.0, P < 0.05 and FDR < 0.05.

In order to explore whether circRNAs regulates parental gene transcription, Gene Ontology analysis of the genes producing differently expressed circRNAs was performed. Compared to adjacent non-tumorous tissues, the data revealed that the gene expression profile of linear counterparts of differentially over-expressed circRNAs in bladder cancer group favored protein modification process, protein binding and cellular protein metabolic process (Figure 5A), while, Gene ontology enrichment analysis for downregulated transcripts of BC showed that closely related GO terms were molecular function and catalytic activity (Figure 5B). Based on these results, these biological process and molecular functions could contribute to the development of BC.

### Co-expression of lncRNAs/mRNAs and function prediction

**Figure 6:**
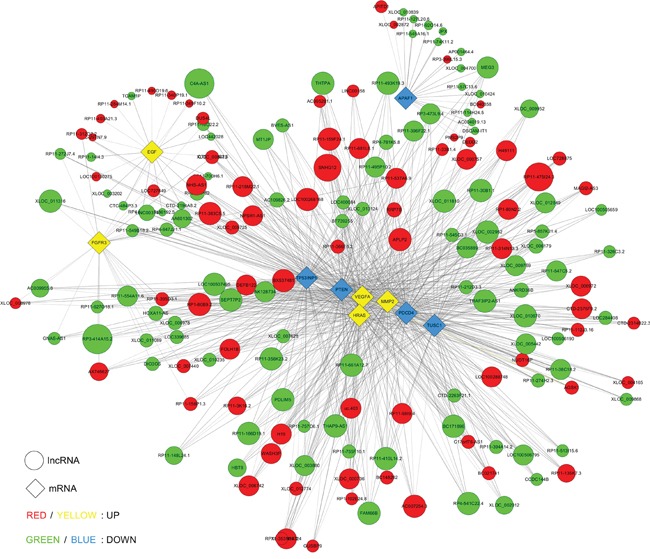
Co-expression network of ten significant mRNAs with their associated lncRNAs The network is based on Pearson correlation coefficient (the absolute value of PCC ≥ 0.90, p-value <0.01 and FDR <0.01), and solid lines mean positive correlations while dashed lines mean negative correlations.

Up to now, functions of most lncRNAs have not been annotated. Therefore, the functional forecast of lncRNAs is according to the annotations of the co-expressed mRNAs function. We chose 10 significantly expressed coding genes in BC to build CNC network according to the degree of correlation (Figure 6). The mRNAs are implicated in a number of biological processes, such as cell cycle, apoptosis, EMT and angiogenesis. The network displayed that upregulated lncRNA APLP2 was negatively and downregulated lncRNA RP11-661A12.7 was positively correlated with PTEN, PDCD4, TUSC1 and TP53INP1, which involved in apoptosis; whereas, upregulated lncRNA RP11-537A6.9 was positively while downregulated lncRNA THAP9-AS1 was negatively correlated with VEGFA, MMP2 and HRAS, which are all associated with metastasis. The co-expression network displayed that one mRNA or lncRNA may correlate with one to dozens of lncRNAs. The co-expression network might suggest that the regulation between lncRNAs and mRNAs is implicated in BC.

### *Cis* and *trans* regulating function prediction of lncRNAs (lncRNAs' nearby coding genes and lncRNA-TF-mRNA)

**Figure 7:**
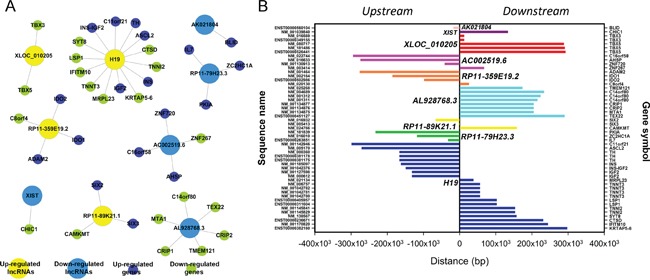
*Cis* regulation of lncRNAs to nearby coding genes **A.** LncRNAs and their potential *cis* regulated nearby genes are shown in the network. The large yellow nodes represent the up-regulated lncRNAs and the large blue nodes represent the down-regulated lncRNAs. The tiny dark blue nodes mean the up-regulated genes and the tiny green nodes mean the down-regulated genes. **B.** The distances between lncRNAs and their *cis* regulated genes are presented. The left vertical axis shows the sequence name of coding genes, and the right vertical axis displays gene symbol of the sequence.

Next, according to co-expression, we further detected how the dysregulated lncRNAs might play a cis or trans-regulatory role in mRNA genes. A correlated expression networks were built to search the underlying relation of the 10 lncRNAs and adjacent coding gene. We chose the top 10 most significantly differentially expressed lncRNAs to hunt their nearby coding genes. The co-expressed protein-coding genes were defined as cis-regulated genes with one differentially expressed lncRNA within 300 kb on the same chromosome. Each lncRNA has a different number of neighboring coding genes. For example, H19 had maximum number of 14 adjacent coding genes, whereas XIST had only 1 nearby coding gene. LncRNAs RP11-359E19.2 and RP11-79H23.3 had 4 and 3 nearby coding genes respectively (Figure 7). The networks could furnish valuable clue for these lncRNAs with nearby coding genes.

**Figure 8:**
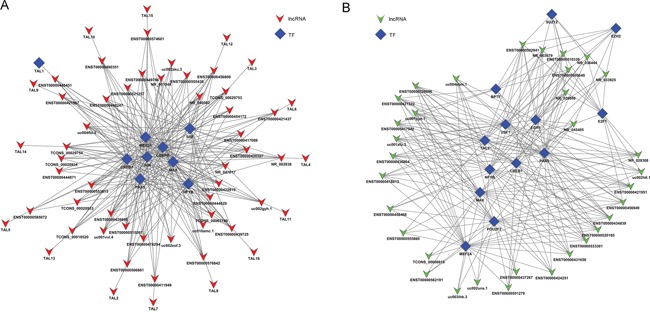
The network of enrichment transcription factors and quary lncRNAs **A.** The up-regulated lncRNAs - TFs network consist of 9 TFs and correlated 56 lncRNAs, and they are connected by 261 edges. **B.** The up-regulated lncRNAs - TFs network consist of 13 TFs and correlated 33 lncRNAs, and they are connected by 232 edges.

**Figure 9:**
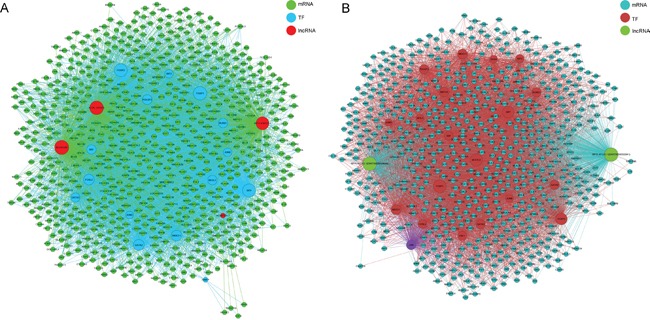
The lncRNA-TFs-genes *trans* regulation network **A.** The up-regulated lncRNAs-TFs-genes network consist of 3 lncRNAs, 15 TFs and correlated 737 genes. **B.** The down-regulated lncRNAs-TFs-genes network consist of 2 lncRNAs, 20 TFs and correlated 662 genes.

To explore the role of lncRNAs in BC, we searched the TF correlated with lncRNAs according to the enrichment with cumulative hypergeometric test, and then, a co-expression network of combining differentially expressed lncRNAs with TF was constructed. The trans-regulatory functions of lncRNAs were predicted by the TFs that could regulate their expression. Some lncRNAs might take part in particular pathways regulated by TFs. Therefore, supposing lncRNAs could have trans-regulatory functions, we analyzed the co-expressed mRNAs with these lncRNAs and mRNAs regulated by TFs. Using the threshold of P < 0.01 and FDR <0.01, each lncRNA could connect with one to more than a dozen TFs and each pair of lncRNA-TF is the result of several genes enrichment (Figure 8), which provided key data for subsequent research. Upregulated 50 lncRNAs were found corresponding to 9 TFs, while downregulated 50 lncRNA were discovered corresponding to 13 TFs. Then, we further introduced mRNAs to build the TF-lncRNA-mRNA ternary network on the base of TF-lncRNA binary analyses (Figure 9). We found that most of lncRNAs participate in pathways regulated by TFs: JUN, MAX, EZH2, EGR1, PAXS, USF1, CREB1, JUN and SFR, etc, suggesting that these TFs could be correlated with tumorigenesis and development of bladder cancer.

### Construction of ceRNA network

**Figure 10:**
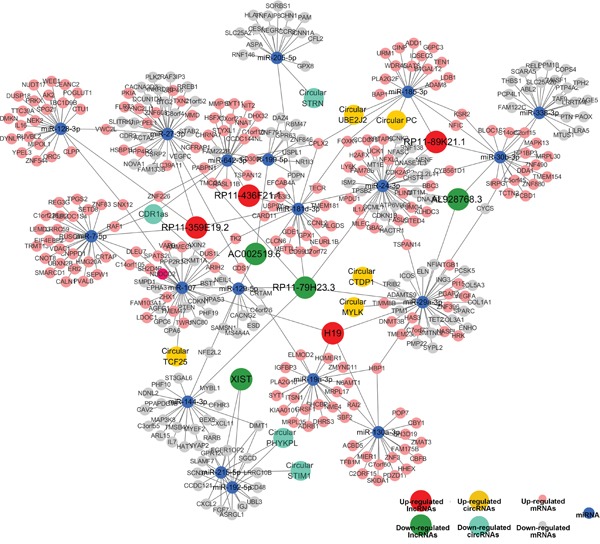
Competing endogenous RNA network in bladder carcinoma The competing endogenous RNA network has been based on lncRNA/miRNA, circRNA/miRNA and miRNA/mRNA interactions. In this network, the edges represent sequence matching, and lncRNAs or circRNAs connects expression correlated mRNAs via miRNAs.

According to ceRNA hypothesis, competing endogenous RNAs (ceRNAs) members can compete for the same MREs to regulate each other. RNA transcripts communicate through the ceRNA language. We pioneered a ceRNA network in bladder cancer by our microarray data (Figure 10). We selected differentially expressed 8 lncRNAs and 9 circRNAs, sharing a common binding site of MRE. For instance, lncRNA H19, circular MYLK and circular CTDP1 are ceRNA of miR-29a-3p targeting DNMT3B, ITGB1, VEGFA and HAS3. Whereas, lncRNA RP11-89K21.2 and circular PC are ceRNA of miR-185-3p targeting ADD1and BAP1. These RNA interactions would supply novel perspective for the tumorigenesis mechanisms of BC.

## DISCUSSION

The conventional view of gene regulation focused on protein-coding genes until the discovery of numerous non-coding RNAs including lncRNAs and circRNAs. Especially, some researches of deregulated lncRNA expression covering many kinds of cancer suggest that abnormal lncRNA expression might contribute to tumorigenesis and progression [12, 13]. More recently, circRNAs also reveal the potential roles in cancer [14]. However, comprehensive analyses of differentially expressed profiles of lncRNAs and circRNAs in BC have not yet been reported. To probe the functions of lncRNAs and circRNAs in bladder carcinoma tumors, here, we explored expression profiles of lncRNAs and circRNAs in the genome-wide for 4 bladder cancer and matched adjacent tissues using microarray.

We gain a landscape of lncRNA and circRNAs expression by microarray. A total of 2045 and 285 up as well as 2110 and 184 down regulated lncRNAs and circRNAs were identified to reveal significantly differential expression in BC, respectively. Approximately 60% deregulated lncRNAs belongs to intergenic. Five deregulated lncRNAs and four mRNAs were further verified using qRT-PCR. Mainly, the qRT-PCR data coincided with microarray assays. These differentially expressed genes were subsequently integrated into hierarchical categories according to heat map and hierarchical clustering. We also showed that differentially expressed lncRNAs were distributed on each chromosome, which suggested that each chromosome, including the X and Y chromosomes, is associated with various degrees of abnormality in BC tumorigenesis. In accordance with previous researches, our data also revealed a 30 fold upregulation of H19 in BC. It has been known that H19 is a potent oncogene, and it is associated with tumorigenesis, metastasis and poor prognosis of bladder cancer [15, 16]. In particular, our data also indicated 7 fold decreasing of AY343891 in tumor tissues, which involved in tumorigenesis of BC [17]. LncRNAs, a new kind regulators of gene expression, are now coming out. The expression profile of lncRNAs in BC was investigated. In the present study, some dysregulated lncRNAs were also detected in other recent studies, which verified these results mutually [18]. However, most differentially expressed lncRNAs have not been studied yet.

KEGG pathway analysis for the differentially expressed mRNAs revealed 20 pathways that could play pivotal roles in tumorigenesis mechanisms of BC including p53, bladder cancer, cell cycle, chemical carcinogenesis and Cytokine-cytokine receptor interaction in BC group compared with the normal control, suggesting that dyregulated mRNAs may play crucial role for these targets through regulating associated pathways in BC. Our result support Zhu et al' finding [19]. The annotation results of the most significant Gene Ontology items were cellular metabolic process, poly(A) RNA binding, immune system process, immune response and receptor binding, indicating related coding gene contribute to development of BC. Interestingly, the top 4 decreased biological processes belong to immune, it is further confirmed that the decline of the immune system function is closely correlative to tumorigenesis [20].

A correlation between circRNAs expression and expression of their linear counterparts were performed using Gene Ontology analysis. GO terms showed that some biological process and molecular functions could involve in the development of BC. It has been reported that circular RNAs could play a role as microRNA sponges. 85% of circular RNAs are aligned in sense orientation to known protein-coding genes, and they span 1–5 exons. Recently, circular RNAs were proposed to harbor microRNAs (miRNAs), and were found to be enriched with functional miRNA binding sites. Li et al demonstrated that Cir-ITCH could act as a miRNA sponge and increase expression of the miRNA target gene ITCH in esophageal squamous cell carcinoma [21]. However, it has been reported that the biogenesis of circRNA could compete with the splicing of pre-mRNA, which suggests that the biogenesis of circRNA might play an important role in producing mRNA [22]. Whether circRNA affects the expression of linear mRNA needs further study in the future.

To date, the functions of most lncRNAs are not well understood. Constructing CNC co-expression network was a means for the prediction of lncRNAs function [23]. We also found that many lncRNA expression levels were significantly correlated with the expression of dozens of protein coding genes. Therefore, a CNC network was built to further survey the relation of dyregulated lncRNAs and mRNAs. Some major connected mRNAs have been known to be associated with BC, such as VEGFA, PTEN, EGF and FGFR3 [24–26]. We thus deduced that these lncRNAs might be correlated with carcinogenesis of BC by regulating co-expression genes.

The research reported that lncRNAs could function to impact on transcription of neighboring or remote genes through cis- and trans-regulatory mechanisms [27]. Next, a network of the top 10 significantly dysregulated lncRNAs with their adjacent coding genes was delineated, which might provide new clue for elucidating the underlying mechanism of BC. As shown in the Figure 7, the chromosomal location of H19 is 11p15.5, there are 14 annotated neighboring coding genes near it. H19 reciprocally imprinted and regulated its adjacent gene IGF2. Accumulating evidences show that H19 might play pivotal roles in metastasis via the epithelial to mesenchymal (EMT) and the mesenchymal to epithelial transitions (MET) [28]. Intriguingly, 6 nearby coding genes corresponding to downregulated AL928768.3 were all downregulated, which displayed its positive regulatory role at transcriptional level; whereas, 3 coding genes IL7, PKIA and ZC2HC1 neighbouring downregulated RP11-79H23.3A were all upregulated, suggesting RP11-79H23.3A has a tumor suppressor function in BC.

Another constructed network in the current study was lncRNAs-TFs network. It has been reported that some lncRNAs were regulated by TFs [29]. TFs combining cis-elements can also regulate expression of target gene at promoter location [30]. It has been known that some TFs involved in BC pathogenesis. Therefore, compositive analysis of TFs and differential co-expression genes might provide better comprehension on pathogenesis of BC. JUN, MAX, NFYB, EGR1, PAX5, CEBPB, CREB1, MEF2A, and SRF were the most relevant TFs. The researches demonstrated that some of them were associated with BC [31–33], but, the underlying mechanisms remain unknown. Therefore, we hypothesized that these TFs could play vital roles in the tumorigenesis of BC through regulating the lncRNAs and mRNAs. C-jun is thought to be an essential component of the transcription factor AP-1 that is critical for proliferation, differentiation and apoptosis [34]. Max is a multifunctional transcription factor, which forms a heterodimer with transcription factor C-Myc or Mad, which is combined with the E-box on the target gene promoter to regulate the expression of the target gene [35]. However, the relationship between lncRNAs-TFs needs to be further investigated.

Researches have shown that ceRNAs have an important influence on regulating gene expression at post-transcriptional level and are involved in oncogenesis and cancer progression. More than 25,000 circular RNAs have been identified in human. Recently, circular RNAs were proposed to harbor microRNAs (miRNAs), and were found to be enriched with functional miRNA binding sites. To date, there has been no report on ceRNAs in BC. Here, for the first time, we constructed a lncRNA-miRNA-circRNA-mRNA ceRNA network of bladder cancer based on our microarray data. Recently, several reports revealed that some lncRNAs might implicate in some cancer as ceRNAs [36–38]. However, the functions of lncRNAs and circRNAs associated-ceRNAs in tumorigenesis are not fully understood. The results showed a specific BC-associated ceRNA network. 8 lncRNAs (H19, RP11-89K21.1 and RP11-436FK21.1, etc), 9 circRNAs (CDR1as, circular PC and circular MYLK, etc) and 19 miRNAs (miR-29a-3p, miR-24-3p, miR-128-3p, miR-7-5p, miR-107, etc) were involved. For instance, we focused on miRNA-29a-3p, as a suppressor onco-miRNA. lncRNA-H19 and circular MYLK as well as circular CTDP1 could regulate the expression of DNMT3B, HAS3, VEGFA and ITGB1 through competing miRNA response elements (MREs) of miRNA-29a-3p, which would result in growth and metastasis of cancer. These data suggest that lncRNAs and circRNAs harbor MREs and play pivotal regulating roles in BC. These pioneering discoveries might enrich our understanding of the pathogenesis of BC and provide novel clinical marker for BC. Further research on ceRNAs of miRNA-29a-3p and other associated functions are being carried out in our laboratory.

To conclude, we found a profile of dysregulated lncRNAs and circRNAs that might be prospective clinical markers and associated with tumorigenesis and development of BC. Our data might lay a foundation for further functional research of lncRNAs and circRNAs in BC. These results suggest that specific lncRNAs and circRNAs could be valuable for diagnosis and therapy of bladder cancer and be of biological importance.

## MATERIAL AND METHODS

### Patients and samples

The research protocol was approved and supervised by The Ethics Committee of Chongqing Medical University. Up to date, we have collected 30 pairs of bladder carcinoma and para-carcinoma tissues from patients who went through surgical treatment without preoperative chemotherapy or radiotherapy in The First Affiliated Hospital of Chongqing Medical University. All the patients gave their written informed consent. Samples were pathologically confirmed, and were stored in liquid nitrogen immediately after surgical resection.

### RNA extraction

Total RNA was extracted from bladder carcinoma and para-carcinoma tissues using TRIzol (Invitrogen, USA) according to manufacturer's instructions. Subsequently, total RNA was assessed by electrophoresis on a denaturing agarose gel and quantified by NanoDrop spectrophotometer (NanoDrop, USA).

### Microarray assay

Four pairs of carcinoma and para-carcinoma tissues were used for microarray assay to determine differentially expressed lncRNA, circRNA and mRNAs comparing bladder cancer and normal control. The microarray hybridization was performed based on the manufacturer's standard protocols (Agilent Technology) including purifying RNA, transcribing into fluorescent cRNA, and then hybridizing onto the Human lncRNA Array v3.0 (Arraystar) and Human circRNA Arrays (Arraystar). Finally, the hybridized slides were washed, fixed and scanned to images by the Agilent Scanner G2505C. And the data collection was performed using Agilent Feature Extraction software (version 11.0.1.1). The raw data were quantile normalized and further data analysis was performed with R software package, GeneSpring GX (Agilent Technologies) and gene expression dynamics inspector (GEDI). The statistical significance of differentially regulated lncRNAs, circRNAs and mRNAs between bladder carcinoma group and normal control group was identified through p-value and FDR filtering. Significant differential expressed transcripts were retained by screening fold change ≥2.0, P < 0.05 and FDR <0.05. Hierarchical clustering was performed to generate an overview of the characteristics of expression profiles based on values of all expressed transcripts and significant differential expressed transcripts.

### Correlation and co-expression analysis

The co-expression analysis was based on calculating the Pearson correlation coefficient (PCC) between coding genes and noncoding transcripts according to their expression levels. The absolute value of parameter PCC ≥0.90, p-value <0.01 and FDR <0.01 was recommended and retained for further analysis.

### Quantitative real-time PCR validation

30 pairs of bladder carcinoma tissues samples and normal tissues were used for qRT-PCR validation. After RNA isolation, M-MLV reverse transcriptase (Invitrogen, USA) was used for synthesizing cDNA according to the manufacturer's instructions. Subsequently, we performed qRT-PCR using SYBR Green assays in a total reaction volume of 10 μl, including 0.5 μl PCR Forward Primer (10 μM), 0.5 μl PCR Reverse Primer (10 μM), 2 μl cDNA, 5 μl 2×Master Mix and 2 μl double distilled water. The protocol was initiated at 95°C for 10min, then at 95°C (10 sec), 60°C (60 sec) for a total 40 cycles. β-actin was used as a reference. Results were harvested in three independent wells. For quantitative results, the relative expression level of each circRNA was calculated using 2^−ΔΔCt^ method. Student's t-tests were applied and p-value < 0.05 was considered to be significant. The values were expressed as means ± SD. The primers are below. RP11-359E19.2: F, 5′TTTCCCTTCTCAACGACCTG3′; R, 5′GAGTAAAGACAAGCTGAACCCA3′. AL928768.3: F, 5′AGATGCCGCCTCCCTATGT3′; R, 5′CACGGTCAGTGCTGTGCTAT3′. AC002519.6: F, 5′TGCTCATTTCACTCCACCCA3′; R, 5′ATTTCCTTTGCCTTCTCGC3′. RP11-79H23.3: F, 5′GCAAGGAGAGTAATGCTGGA3′; R, 5′CAATGAGGATGAGAAGAGGTC3′. AK021804: F, 5′GGTTTCTCAACACCCCTTCTTC3′; R, 5′CCCCAGTTCCCCGCCTTAT3′. HRAS: F, 5′CAGTACAGGGAGCAGATCAAACG3′; R, 5′TGAGCCTGCCGAGATTCCA3′. VEGFA: F, 5′CATGCAGATTATGCGGATCAA3′; R, 5′ GCATTCACATTTGTTGTGCTGTAG3′. ITGB1: F, 5′GAATGCCTACTTCTGCACGATGT3′; R, 5′TGTTCCTTTGCTACGGTTGGTTA3′. DNMT3B: F, 5′AAGAGTTGGGCATAAAGGTAGGA3′; R, 5′GGCTGGATTCACATTTGAGAGA3′. β-actin: F, 5′CCTGTACGCCAACACAGTGC3′; R, 5′ATACTCCTGCTTGCTGATCC3′.

### Competing endogenous RNA network analysis

Those lncRNAs/circRNAs and mRNAs whose expression levels shared a meaningful correlation were subjected to the analysis. The potential miRNA response elements were searched on the sequences of lncRNAs/circRNAs and mRNAs, and the overlapping of the same miRNA seed sequence binding site both on the lncRNAs/circRNAs and the mRNA predicted lncRNA/circRNA-miRNA-mRNA interaction. The miRNA binding sites were predicted by miRcode (http://www.mircode.org/), while the miRNA-mRNA interactions were predicted by Targetscan (http://www.targetscan.org/).

### Gene ontology (GO) and pathway analysis

We conducted Gene Ontology (GO) analysis (http://www.geneontology.org) to construct meaningful annotation of genes and gene products in a wide variety of organisms. The ontology has covered domains of biological processes, cellular components and molecular functions. The-log10 (p-value) denotes enrichment score representing the significance of GO term enrichment among differentially expressed genes. We also performed KEGG pathway analysis to harvest pathway clusters covering our knowledge on the molecular interaction and reaction networks in differentially regulated gene profiling. Also the −log10 (p-value) denotes enrichment score showing the significance of the pathway correlations.

### *Cis* and *trans* regulation prediction

Previous study defined that a cis-regulator is the one that exerts its function on a neighbouring gene located at the same chromosome, and a trans-regulator is the one that does not meet the criterion. And lncRNAs are found to regulate gene expressions both on cis and trans manner. We subjected the significantly changed lncRNAs whose expression levels were correlated with that of mRNAs to cis and trans prediction. For cis prediction, we identified genomic localization of the paired lncRNAs and mRNAs. As a result, the nearby gene that is less than 300kb upstream or downstream away from the lncRNA can be the potential target regulated by that lncRNA in cis manner. For trans prediction, we focused on the manner that lncRNAs play their functions via transcription factors (TFs). Therefore, we enriched those mRNAs co-expressed with lncRNAs significantly overlapped with the target genes of a given TF and constructed the lncRNAs-TFs-mRNAs network. The enrichment and connectivity was based on Position Frequency Matrix (PFM) performed as described previously [39, 40].

## References

[1] Zhao F, Lin T, He W, Han J, Zhu D, Hu K, Li W, Zheng Z, Huang J, Xie W (2015). Knockdown of a novel lincRNA AATBC suppresses proliferation and induces apoptosis in bladder cancer. Oncotarget.

[2] Zhuang J, Lu Q, Shen B, Huang X, Shen L, Zheng X, Huang R, Yan J, Guo H (2015). TGFβ1 secreted by cancer-associated fibroblasts induces epithelial-mesenchymal transition of bladder cancercells through lncRNA-ZEB2NAT. Sci Rep.

[3] Chen Z, Luo Y, Yang W, Ding L, Wang J, Tu J, Geng B, Cui Q, Yang J (2015). Comparison Analysis of Dysregulated LncRNA Profile in Mouse Plasma and Liver after HepaticIschemia/Reperfusion Injury. PLoS One.

[4] He Y, Meng XM, Huang C, Wu BM, Zhang L, Lv XW, Li J (2014). Long noncoding RNAs: Novel insights into hepatocelluar carcinoma. Cancer Lett.

[5] Lee S, Kopp F, Chang TC, Sataluri A, Chen B, Sivakumar S, Yu H, Xie Y, Mendell JT (2016). Noncoding RNA NORAD Regulates Genomic Stability by Sequestering PUMILIO Proteins. Cell.

[6] Xue Y, Ma G, Zhang Z, Hua Q, Chu H, Tong N, Yuan L, Qin C, Yin C, Zhang Z, Wang M (2015). A novel antisense long noncoding RNA regulates the expression of MDC1 in bladder cancer. Oncotarget.

[7] Hansen TB, Jensen TI, Clausen BH, Bramsen JB, Finsen B, Damgaard CK, Kjems J (2013). Natural RNA circles function as efficient microRNA sponges. Nature.

[8] Guo JU, Agarwal V, Guo H, Bartel DP (2014). Expanded identification and characterization of mammalian circular RNAs. Genome Biol.

[9] Wang YH, Yu XH, Luo SS, Han H (2015). Comprehensive circular RNA profiling reveals that circular RNA100783 is involved in chronic CD28-associated CD8(+)T cell ageing. Immun Ageing.

[10] Salmena L, Poliseno L, Tay Y, Kats L, Pandolfi PP (2011). A ceRNA hypothesis: the Rosetta Stone of a hidden RNA language?. Cell.

[11] Peng H, Lu M, Selaru FM (2015). The genome-wide gene expression profiling to predict competitive endogenous RNA network in hepatocellular cancer. Genom Data.

[12] Taniue K, Kurimoto A, Sugimasa H, Nasu E, Takeda Y, Iwasaki K, Nagashima T, Okada-Hatakeyama M, Oyama M, Kozuka-Hata H, Hiyoshi M, Kitayama J, Negishi L, Kawasaki Y, Akiyama T (2016). Long noncoding RNA UPAT promotes colon tumorigenesis by inhibiting degradation of UHRF1. Proc Natl Acad Sci U S A.

[13] Sahu A, Singhal U, Chinnaiyan AM (2015). Long noncoding RNAs in cancer: from function to translation. Trends Cancer.

[14] Qin M, Liu G, Huo X, Tao X, Sun X, Ge Z, Yang J, Fan J, Liu L, Qin W (2016). Hsa_circ_0001649: A circular RNA and potential novel biomarker for hepatocellular carcinoma. Cancer Biomark.

[15] Luo M, Li Z, Wang W, Zeng Y, Liu Z, Qiu J (2013). Long non-coding RNA H19 increases bladder cancer metastasis by associating with EZH2 and inhibiting E-cadherin expression. Cancer Lett.

[16] Luo M, Li Z, Wang W, Zeng Y, Liu Z, Qiu J (2013). Upregulated H19 contributes to bladder cancer cell proliferation by regulating ID2 expression. FEBS J.

[17] Luo H, Zhao X, Wan X, Huang S, Wu D (2014). Gene microarray analysis of the lncRNA expression profile in human urothelial carcinoma of the bladder. Int J Clin Exp Med.

[18] Peter S, Borkowska E, Drayton RM, Rakhit CP, Noon A, Chen W, Catto JW (2014). Identification of differentially expressed long noncoding RNAs in bladder cancer. Clin Cancer Res.

[19] Zhu YP, Bian XJ, Ye DW, Yao XD, Zhang SL, Dai B, Zhang HL, Shen YJ (2014). Long noncoding RNA expression signatures of bladder cancer revealed by microarray. Oncol Lett.

[20] Bigley AB, Spielmann G, LaVoy EC, Simpson RJ (2013). Can exercise-related improvements in immunity influence cancer prevention and prognosis in the elderly?. Maturitas.

[21] Li F1, Zhang L2, Li W1, Deng J1, Zheng J1, An M1, Lu J3, Zhou Y (2015). Circular RNA ITCH has inhibitory effect on ESCC by suppressing the Wnt/β-catenin pathway. Oncotarget.

[22] Ashwal-Fluss R, Meyer M, Pamudurti NR, Ivanov A, Bartok O, Hanan M, Evantal N, Memczak S, Rajewsky N, Kadener S (2014). circRNA biogenesis competes with pre-mRNA splicing. Mol Cell.

[23] Zhao F, Qu Y, Liu J, Liu H, Zhang L, Feng Y, Wang H, Gan J, Lu R, Mu D (2015). Microarray Profiling and Co-Expression Network Analysis of LncRNAs and mRNAs in Neonatal Rats Following Hypoxic-ischemic Brain Damage. Sci Rep.

[24] Gao Y, Wu K, Chen Y, Zhou J, Du C, Shi Q, Xu S, Jia J, Tang X, Li F, Hui K, He D, Guo P (2015). Beyond proliferation: KLF5 promotes angiogenesis of bladder cancer through directly regulatingVEGFA transcription. Oncotarget.

[25] Lei M, Xie W, Sun E, Sun Y, Tian D, Liu C, Han R, Li N, Liu M, Han R, Liu L (2015). microRNA-21 Regulates Cell Proliferation and Migration and Cross Talk with PTEN and p53 in Bladder Cancer. DNA Cell Biol.

[26] Chang CH, Chan PC, Li JR, Chen CJ, Shieh JJ, Fu YC, Chen HC, Wu MJ (2015). Gab1 is essential for membrane translocation, activity and integrity of mTORCs after EGF stimulation in urothelial cell carcinoma. Oncotarget.

[27] Xiong W, Jiang YX, Ai YQ, Liu S, Wu XR, Cui JG, Qin JY, Liu Y, Xia YX, Ju YH, He WJ, Wang Y, Li YF, Hou Y, Wang L, Li WH (2015). Microarray Analysis of Long Non-coding RNA Expression Profile Associated with 5-Fluorouracil-Based Chemoradiation Resistance in Colorectal Cancer Cells. Asian Pac J Cancer Prev.

[28] Raveh E, Matouk IJ, Gilon M, Hochberg A (2015). The H19 Long non-coding RNA in cancer initiation, progression and metastasis - a proposed unifying theory. Mol Cancer.

[29] Wang Y, Qian CY, Li XP, Zhang Y, He H, Wang J, Chen J, Cui JJ, Liu R, Zhou H, Xiao L, Xu XJ, Zheng Y, Fu YL, Chen ZY, Chen X, Zhang W, Ye CC, Zhou HH, Yin JY, Liu ZQ (2015). Genome-scale long noncoding RNA expression pattern in squamous cell lung cancer. Sci Rep.

[30] Tian L, Cao J, Deng X, Zhang C, Qian T, Song X, Huang B (2014). Unveiling transcription factor regulation and differential co-expression genes in Duchenne muscular dystrophy. Diagn Pathol.

[31] Yuan F, Xu Z, Yang M, Wei Q, Zhang Y, Yu J, Zhi Y, Liu Y, Chen Z, Yang J (2013). Overexpressed DNA polymerase iota regulated by JNK/c-Jun contributes to hypermutagenesis in bladder cancer. PLoS One.

[32] Jeong KC, Kim KT, Seo HH, Shin SP, Ahn KO, Ji MJ, Park WS, Kim IH, Lee SJ, Seo HK (2014). Intravesical instillation of c-MYC inhibitor KSI-3716 suppresses orthotopic bladder tumor growth. J Urol.

[33] Li CF, Wu WJ, Wu WR, Liao YJ, Chen LR, Huang CN, Li CC, Li WM, Huang HY, Chen YL, Liang SS, Chow NH, Shiue YL (2015). The cAMP responsive element binding protein 1 transactivates epithelial membrane protein 2, a potential tumor suppressor in the urinary bladder urothelial carcinoma. Oncotarget.

[34] Xu L, Ning H, Gu L, Wang Q, Lu W, Peng H, Cui W, Ying B, Ross CR, Wilson GM, Wei L, Wold WS, Liu J (2015). Tristetraprolin induces cell cycle arrest in breast tumor cells through targeting AP-1/c-Jun and NF-κB pathway. Oncotarget.

[35] Lüscher B (2001). Function and regulation of the transcription factors of the Myc/Max/Mad network. Gene.

[36] Qu J, Li M, Zhong W, Hu C (2015). Competing endogenous RNA in cancer: a new pattern of gene expression regulation. Int J Clin Exp Med.

[37] Peng W, Si S, Zhang Q, Li C, Zhao F, Wang F, Yu J, Ma R (2015). Long non-coding RNA MEG3 functions as a competing endogenous RNA to regulate gastric cancer progression. J Exp Clin Cancer Res.

[38] Xia T, Liao Q, Jiang X, Shao Y, Xiao B, Xi Y, Guo J (2014). Long noncoding RNA associated-competing endogenous RNAs in gastric cancer. Sci Rep.

[39] Fu M, Huang G, Zhang Z, Liu J, Zhang Z, Huang Z, Yu B, Meng F (2015). Expression profile of long noncoding RNAs in cartilage from knee osteoarthritis patients. Osteoarthritis Cartilage.

[40] Guttman Mitchell, Rinn John L (2012). Modular regulatory principles of large non-coding RNAs. Nature.

